# Evaluating the Cost-Effectiveness of Pre-Exposure Prophylaxis (PrEP) and Its Impact on HIV-1 Transmission in South Africa

**DOI:** 10.1371/journal.pone.0013646

**Published:** 2010-11-05

**Authors:** Carel Pretorius, John Stover, Lori Bollinger, Nicolas Bacaër, Brian Williams

**Affiliations:** 1 Futures Institute, Glastonbury, Connecticut, United States of America; 2 IRD (Institut de Recherche pour le Developpement), Bondy, France; 3 South African Centre for Epidemiological Modelling and Analysis (SACEMA), DST/NRF Centre of Excellence in Epidemiological Modelling and Analysis, Stellenbosch University, Stellenbosch, South Africa; University of Cape Town, South Africa

## Abstract

**Background:**

Mathematical modelers have given little attention to the question of how pre-exposure prophylaxis (PrEP) may impact on a generalized national HIV epidemic and its cost-effectiveness, in the context of control strategies such as condom use promotion and expanding ART programs.

**Methodology/Principal Findings:**

We use an age- and gender-structured model of the generalized HIV epidemic in South Africa to investigate the potential impact of PrEP in averting new infections. The model utilizes age-structured mortality, fertility, partnership and condom use data to model the spread of HIV and the shift of peak prevalence to older age groups. The model shows that universal PrEP coverage would have to be impractically high to have a significant effect on incidence reduction while ART coverage expands. PrEP targeted to 15–35-year-old women would avert 10%–25% (resp. 13%–28%) of infections in this group and 5%–12% (resp. 7%–16%) of all infections in the period 2014–2025 if baseline incidence is 0.5% per year at 2025 (resp. 0.8% per year at 2025). The cost would be $12,500–$20,000 per infection averted, depending on the level of ART coverage and baseline incidence. An optimistic scenario of 30%–60% PrEP coverage, efficacy of at least 90%, no behavior change among PrEP users and ART coverage less than three times its 2010 levels is required to achieve this result. Targeting PrEP to 25–35-year-old women (at highest risk of infection) improves impact and cost-effectiveness marginally. Relatively low levels of condom substitution (e.g., 30%) do not nullify the efficacy of PrEP, but reduces cost-effectiveness by 35%–40%.

**Conclusions/Significance:**

PrEP can avert as many as 30% of new infections in targeted age groups of women at highest risk of infection. The cost-effectiveness of PrEP relative to ART decreases rapidly as ART coverage increases beyond three times its coverage in 2010, after which the ART program would provide coverage to more than 65% of HIV_+_ individuals. To have a high relative cost-effective impact on reducing infections in generalized epidemics, PrEP must utilize a window of opportunity until ART has been scaled up beyond this level.

## Introduction

Antiretroviral therapy (ART) forms the basis of many HIV-related treatment and prophylactic strategies [1]. Combination therapies have prevented progression to AIDS and have reduced mortality in many HIV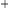 individuals [2], [3]. The use of antiretroviral regimens such as zidovudine and nevirapine has been very effective in preventing transmissions (pre- and post-partum) from mother to child [4]. Post-exposure prophylaxis (PEP) using mostly zidovudine is recommended for individuals following recognized recent exposure to HIV from unprotected sex or needle use or accidental exposure during health care. Policy makers and researchers are now investigating ways to extend the use of antiretroviral therapy to limit the spread of HIV at population level.

Pre-exposure prophylaxis (PrEP) advocates the use of antiretroviral therapy by individuals who anticipate exposure to HIV infection, including commercial sex workers (CSW), men who have sex with men (MSM), and serodiscordant couples. Although envisaged as effective protection for both men and women at risk, it provides a promising and timely female-controlled strategy for women at high risk [5].

Tenofovir disoproxyl fumarate (TDF) and a combination of TDF and emtricitabine (FTC) are the focus of ongoing PrEP trials. Their safety for treating infection within HIV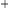 individuals has been well established [6]. Safety trials in Cameroon, Ghana, and Nigeria showed that once-daily oral TDF was well tolerated by HIV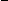 participants over the course of their study participation. However, there are various documented concerns regarding the use of TDF and FTC in PrEP programs. These include the possible emergence of drug-resistance and activity of TDF against liver function and its consequences for those harboring the hepatitis B virus (HBV) [7].

Results from the CAPRISA 004 trial, announced at the 2010 AIDS conference in Vienna [8], indicate that TDF-based microbicide gel is 39% effective in preventing HIV transmission in women [9]. A 54% reduction in new infections was observed among women with high gel adherence. The results hold promise for various PrEP trials that are underway to test the safety and efficacy of TDF (and likely also for FTC) when used as a prevention tool. The ongoing VOICE trial conducted in Uganda, South Africa, Zambia, Zimbabwe and Malawi is a randomized control trial comparing three oral groups (tenofovir, emtricitabine/tenofovir, and placebo) with two topical groups (tenofovir gel vs placebo gel). It will be a key test of the safety and efficacy findings of the CAPRISA 004 trial. The iPrEx trials in Brazil, Ecuador, Peru, South Africa, Thailand and the US, and FEM-PrEP trials in Kenya, Malawi, South Africa, Tanzania and Zambia are also notable [10]. Assuming that all trials qualify to advance to their final stages, i.e. if safety concerns do not emerge and if the protective effect of PrEP is established during early stages of these trials, it will take a number of years until guidelines for the clinical use of PrEP are formulated.

In the meantime millions of new infections will arise, at least in sub-Saharan Africa (SSA). An incidence projection for the South African HIV epidemic at 2020 shows that without further intervention, and assuming continuation of an optimistically high rate of condom use and expansion of the national ART program, the HIV incidence rate could still be 0.5% per year [11], which translates to more than 200,000 new infections each year after 2020 in South Africa.

The cost-effectiveness of PrEP, a key input to PrEP guidelines, is affected by its eventual coverage and the possibility of emerging resistance patterns, which would require expensive resistance management protocols that are yet to be formulated. Furthermore, the HIV epidemics of SSA countries, where PrEP has the potential to avert millions of new infections (roughly 2.7 to 3.2 million estimated in [12]), are currently undergoing complex transitions. If PrEP is integrated into long-term control strategies for generalized epidemics in SSA, it will be introduced as one component in a toolkit of interventions, each being scaled to achievable coverage. It will be crucial but very difficult to demonstrate the relative impact of PrEP on disease burden and its consequent cost-effectiveness.

Mathematical models have been used to study the potential impact of PrEP at the national level. Abbas et al. [12] studied the potential role of PrEP on the future course of the HIV epidemic in Zambia and Vissers et al. studied the potential impact in Botswana, the Nyanza Province in Kenya and India [5]. To the extent that these models reflect epidemic situations in resource-limited settings, their findings point to a substantial reduction in the number of new infections should PrEP be deployed as a control strategy in SSA. Both papers highlight the interplay between PrEP coverage and behavioral disinhibition as reasons for major concern. In [12] this interplay is studied by creating scenarios of increased sexual activity under PrEP, while [5] also considers a decrease in condom use. Paltiel at al. [13] used a simulation model to assess lifetime infection of high-risk groups in the United States under different PrEP scenarios. Their work shows that cost-effectiveness estimates depend critically on baseline incidence assumptions.

Both [5] and [12] mention the potential impact of expanding ART programs which do not yet provide adequate coverage to individuals who are already infected. The focus of this paper is to evaluate PrEP alongside ART and condom-use interventions, informed by national HIV and demographical surveys. To this end we have developed an age-structured model which is contextualized to the South African epidemic, paying attention to distribution of relative infection risks between age categories. In South Africa, for example, the highest risk category would be 25–35-year-old women (Fig.10 [11]).

## Methods

### Model Structure

The model presented here is an elaboration of an age-structured model for the generalized HIV epidemic in South Africa [11]. It utilizes national surveys in South Africa (2002, 2005 and 2008) to derive parameters and tracks the overall pattern reasonably accurately. The model was specifically designed to reflect an age pyramid, HIV prevalence and overall mortality statistics that would be equal to those of South Africa. In this adaptation, individuals within the model are stratified by age, gender, disease and treatment status: susceptible, receiving PrEP, infected, receiving ART for treatment.

The spread of HIV is mechanistically modelled through stable relationship formation, which is governed by the age-dependent rate at which women change and choose their partners. The model does not contain additional risk structure, although many factors are linked to the rapid spread of HIV in South Africa. These include migrant mine workers and female sex workers [14], high age at first marriage [15] and low levels of male circumcision [16]. These factors are indirectly accounted for by initiating the model with high initial prevalence. The subsequent shaping of the generalized HIV epidemic is adequately modelled by the age-mediated heterogeneities in the model. The model tracks time since infection, but does not incorporate a decline in infectiousness with time since infection, as is the case in many HIV models (e.g. [12]).

More complex HIV models have been developed for the South African HIV epidemic. Among the most notable are those of Johnson et al. that include age, various risk-groups, other STDs, time since infection and other factors [17], [18]. The model presented here captures the epidemic through simpler mechanisms and complexity without significant compromise in fitting age-specific prevalence, mortality, behavioral and other data, including the 2008 population survey [19].

Among other purposes, our model was originally used to study the expanding ART program in South Africa, including a possible expansion toward universal access to testing and treatment (UTT). Our age-structured model (as opposed to models without demographical detail such as the model used in Granich et al. [20]) suggests that a lesser annual testing rate of around 20% of all adults would be sufficient to control the epidemic within a decade after full coverage is reached, a finding also reported in [21]. Here, we extend our focus on the potential impact of UTT on the generalized South African HIV epidemic to the potential impact of PrEP, as well as examining the interplay between the two strategies.

### Introducing PrEP

**Figure 1:**
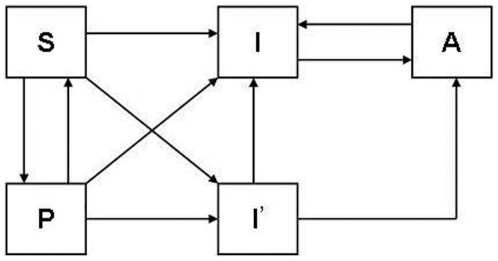
Simple schematic of the model. S-susceptible, I-infected, P-receiving PrEP, I′-infected while on PrEP, A-receiving ART.

The simple addition of a category of people receiving PrEP is shown in Fig. 1 and parameter values are given in Text S1. Susceptible individuals enroll for PrEP at a certain rate. They can acquire infection while on PrEP, depending on its efficacy. A fraction of susceptibles may acquire infection shortly after initiating PrEP. (Here shortly means within one time step of the model, which is one year.) Susceptible PrEP users can ‘drop out’ and discontinue PrEP use. HIV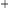 PrEP users discontinue their use of PrEP as a result of regular screening of PrEP users. Those who did not get infected before discontinuing PrEP return to the susceptible category. Those who continue PrEP use will enroll for ART at a (typically) faster rate than they would under the projected testing and treatment rate of the national ART program. The transmission probability of HIV from people on PrEP or ART to susceptible people is assumed to be 10% of that from other HIV_+_ individuals.

TDF and FTC are both susceptible to one-point mutations that can confer resistance [7]. Some researchers are concerned about the possibility that drug-resistance resulting from the mono-therapeutic use of these regimens could subvert ART-based control programs [22]. However, within the relatively simple representation included in [12], drug-resistance did not appear to result from PrEP programs. A limited degree of drug-resistance as a result of PrEP use is also anticipated by [23] and [24]. A recent study investigates the interplay between PrEP use, behavior change, and the transmission of drug-resistant strains [25]. One of its surprising findings is that PrEP targeted to high-risk groups with stable risk behavior could lead to a decrease in HIV transmission and therefore to a decrease in subsequently observed levels of drug-resistant strains. It is also worth noting that the CAPRISA 004 trial reported no detected resistance among seroconvertors using a TDF-based microbicide gel [9].

We avoided the nuances of resistance modelling in order to focus on the cost-effectiveness of PrEP in expanding ART (for treatment) programs. Through regular screening, which is included our model, the possibility of the accumulation in the general population will be reduced.

### Programmatic assumptions

If PrEP is adopted as a control strategy for generalized HIV epidemics, it is likely that the focus will also turn to UTT. Both PrEP (in [12]) and UTT (in [20]) are advocated as cost-effective ways of avoiding large numbers of new infections. These approaches must overcome similar regulatory and programmatic challenges. Although there is currently no advocacy to use PrEP in countries where ART treatment coverage is inadequate, it can certainly become a contentious point, seeing that delivery channels of these two ART-based strategies are likely to overlap.

When PrEP is promoted within certain small high-risk groups it would limit future PrEP and ART delivery tradeoffs of using ART for treatment (UTT) or prevention (PrEP) or both. However, at the national level high-risk groups are both large and difficult to identify, with CSW around mining areas a possible exception. It is therefore reasonable to compare anticipated PrEP coverage scenarios to those of current national ART programs and their potential scale-up. Moreover, the efficacy and relative cost-effectiveness of PrEP will depend on ART coverage at the time when it is introduced.

We make a simple comparison between the coverage and potential impact on HIV incidence of ART and PrEP programs relative to a baseline scenario where ART coverage expands at its current rate. The rate is chosen so that 40% of individuals, who face competing risks of treatment or death, will enroll for ART. This mechanism gave a reasonable fit to the total number of cases receiving ART (Fig.8b [11]). It is assumed that both interventions will start in 2014 and be fully scaled-up by 2019 to achieve a specified testing and enrollment rate. It is assumed that 20% of all confirmed susceptibles older than 15 years will enroll each year for PrEP and that 20% of HIV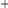 individuals will enroll for ART under a universal test and treat scenario. We assume optimistically, following a recent finding that ART can be 90% effective in preventing transmission [26], that PrEP is 90% effective in preventing infection. We assume an annual drop-out rate of 1.5% for both programs [11].

A key question for PrEP policy is whether coverage could be decreased and the impact of PrEP maintained by targeting groups at highest risk of infection? What is the expected impact of expanding ART coverage on the efficacy of PrEP? We use various scenarios to explore the sensitivity of modelled PrEP impact results with respect to changes in parameter values which reflect PrEP targeting, efficacy and behavioral disinhibition.

The benefits of any new interventions which reduce risk of infection can be negated by risk compensation or behavioral disinhibition, as demonstrated by various male-circumcision impact studies [27], [28]. For example, individuals using PrEP may feel less need to use condoms. On the other hand, levels of condom use may be maintained if PrEP programs include counselling and advocacy to discourage the use of PrEP to substitute the protective effect of condom use [5]. To evaluate the consequence of condom substitution among PrEP users, we use different condom substitution levels in our targeted-PrEP sensitivity analysis.

### Cost assumptions

Our assumptions regarding the cost of PrEP are also kept simple. When TDF is targeted to women this cost could include 1) voluntary counselling (VCT) to limit the number of HIV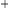 women enrolled for PrEP, 2) various tests, including serum creatinine tests to monitor renal function and detect abnormalities, hepatitis B tests and a pregnancy test, 3) the annual cost of TDF. We make the assumption that PrEP will be available at an annual cost of $150 per year per person. This is based on the following unit costs: $12 annual cost for HIV counselling and testing [29], $4 for serum creatinine testing (the National Health Laboratory Service of South Africa currently perform these at less than $5 per test) and $134 for the PrEP regimen (an optimistic assumption). More frequent testing and failure to negotiate low costs, especially PrEP costs for large-scale PrEP programs, would increase this cost significantly. As in [11], [20] we assume that ART will be available at an average cost (for first and second line therapy) of $600 per year, four times the annual cost of PrEP.

## Results

### Universal PrEP and UTT: comparative impact

To study the impact of PrEP and UTT strategies we constructed four scenarios. In scenario 1 there is no additional ART coverage (i.e. no UTT) and no PrEP. In scenario 2 there is PrEP but no UTT. In scenario 3 there is UTT but no PrEP. In scenario 4 there is both UTT and PrEP. In this comparison PrEP and UTT operates under the programmatic assumptions outlined above.

**Figure 2:**
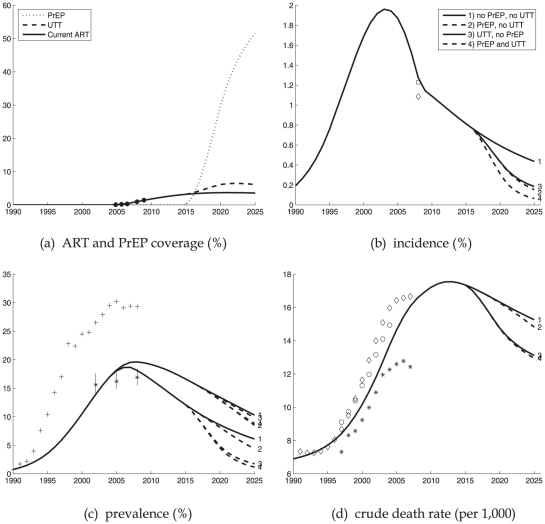
PrEP and UTT: impact and coverage at 2025. (a) Current % of adults on ART (solid line), expanded ART coverage under UTT (dashed line) and PrEP coverage (dotted line). (b) Adult HIV incidence (age 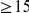) in the model and 2008 UNAIDS (diamond) and ASSA (square) estimates [33]. (c) HIV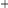 prevalence in antenatal clinics (the upper
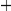
points) as reported in [19] and in the population aged 15–49 (upper solid lines and vertical data points) as reported in [33]. The lower solid line shows the prevalence of HIV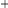 people without ART in the population aged 15–49. (d) Crude death rate. Data from Statistics South Africa (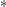) [36], Anderson et al. [37] (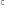), and the US Census Bureau [38] (◊).

Fig. 2a compares the coverage of non-targeted PrEP and UTT strategies in terms of the percentage of the total population enrolled in the respective programs. For the solid line, coverage under the current national ART program expands with no additional intervention. The 2004–2006 data points are from Dorrington et al. [30] and the 2007–2008 data points are estimates provided by WHO, UNAIDS, UNICEF [31]. In 2010 ART coverage would be 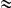
1.4% of the total population, providing ART to 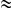
50% of those in need. This is the 2009 coverage estimate reported in [32], which in turn is based on STATSA estimates which are yet to be adjusted to reflect the policy change in eligibility criteria for pregnant women announced in December 2009. In 2025 the ART program, without any additional intervention, will provide coverage to 3.6% of the total population at the current rate of expansion. HIV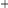 individuals would be infected for an average duration of 9.6 years before initiating ART according to our model.

UTT and PrEP coverage are shown by the dashed and dotted lines respectively. With this UTT program 6.5% of the total population would receive ART by 2025, initiating treatment 6.7 years after infection and reaching more of those in need of treatment. Approximately half of the population in 2006 were adults and close to 20% of these adults were HIV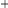 (Fig. 2c). Thus almost 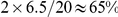 of HIV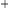 individuals would receive ART. The PrEP program would result in almost all susceptible adults receiving PrEP by 2025 (dotted line), dwarfing the scale of the current ART program.

Fig. 2b shows the potential impact on aggregated HIV incidence in the population aged 15–49. The square (resp. diamond) is the 2008 UNAIDS (resp. ASSA) estimate for the incidence in adults [33]. The data points serve to anchor modelled incidence, which appears to be decreasing, and could reach 0.8% per year by 2014 – less than half of its peak value in 2002. A recent study suggests that incidence among 15–49-year-old men and women was around 2% per year between 2002 and 2005 and declined to 1.3% per year between 2005 and 2008 [34]. A statistically significant decline in incidence of 60% could only be established among 15–24-year-old women during this period. Our baseline incidence curve (upper solid line) agrees with this finding. However, the uncertainty regarding a general incidence decline has bearing on our analysis of the cost-effectiveness of PrEP.

The impact on incidence is shown for each PrEP-ART scenario. The comparison shows that PrEP alone will have a greater impact on incidence than UTT alone. PrEP and UTT together will have the biggest impact.

Fig. 2c shows the potential impact on aggregated HIV prevalence in the population aged 15–49. The antenatal clinic data (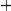
points) for the years 1990–2008 come from the Department of Health of South Africa [33]. The vertical data points for the total adult prevalence come from Shisana et al. [35]. The upper solid shows modelled HIV prevalence for the population aged 15–49. The lower solid line is the projected prevalence of HIV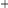 individuals without ART. The PrEP–ART scenarios are shown in dashed lines. The model shows, as is to be expected, no major impact on HIV prevalence by 2025 since HIV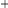 cases receiving treatment would still contribute to prevalence.

Fig. 2d shows the reduction in the crude death rate under each scenario. The 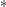 points correspond to registered deaths (Statistics South Africa, [36]), 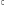 points to estimated deaths according to Anderson et al. [37], and ◊ points to estimates according to the US Census Bureau [38]. Note that the crude death rate has stabilized but that it has not yet been substantially reduced as is expected under ART scale-up [2], [3]. This might be indicating that current ART expansion is still not reaching enough of those in most need in South Africa – a general state of ART programs in resource-limited settings [22].

UTT and similar strategies advocate the use of ART not to address clinical need, but for all individuals found to be HIV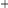 [20], [21]. Frequent testing means ART programs would reach those in most need, averting a substantial number of deaths. Even though PrEP has an impact on incidence, most infections are averted among those facing low risk of mortality. A significant reduction in the crude death rate in the first decade of PrEP (without expanded ART coverage for treatment) is not expected.

### Targeted PrEP: epidemic impact

**Figure 3:**
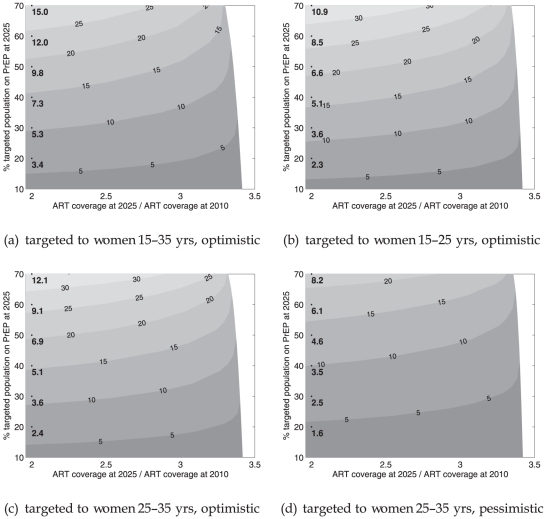
Percentage of new infections (cumulative between 2014 and 2025) averted due to PrEP in targeted group, in addition to infections averted due to ART and condom use. Baseline incidence 0.5% per year at 2025. Contours in intervals of 0%, 5%, 10%, and so on. Vertically spaced points depicts reduction in incidence among all adults. The ordinate corresponds to the 10% intervals of targeted PrEP coverage and the co-ordinate to ART coverage in 2025 reaching twice its 2010 level.

**Figure 4:**
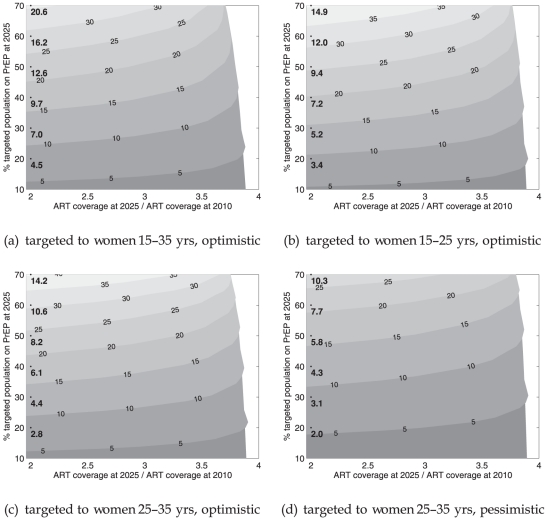
Percentage of new infections (cumulative between 2014 and 2025) averted due to PrEP in targeted group, in addition to infections averted due to ART and condom use. Baseline incidence 0.8% per year at 2025. Contours in intervals of 0%, 5%, 10%, and so on. Vertically spaced points depicts reduction in incidence among all adults. The ordinate corresponds to the 10% intervals of targeted PrEP coverage and the co-ordinate to ART coverage in 2025 reaching twice its 2010 level.

Fig. 3 shows the impact of PrEP in terms of the expected proportional decrease in the number of new infections in the period 2014–2025. We assume that baseline incidence declines as in Fig. 2b (upper solid line). Fig. 4 corresponds to Fig. 3, but assumes a more gradual drop in incidence to 0.8% per year by 2025, based on condom use declining by 15% from 2007 onward. The measure in each figure is shown as a function of the fraction of all adults in the targeted population covered by PrEP at 2025 (vertical axis), ART coverage at 2025 in proportion to ART coverage in 2010 (horizontal axis), using different combinations of efficacy and condom substitution.

The impact among all adults when PrEP is targeted to specific age groups is shown by vertically spaced points corresponding to multiples of 10% in targeted PrEP coverage. Their location on the horizontal axis indicates that ART has reached twice its 2010 coverage by 2025.

In Fig. 3a PrEP initiation is targeted to 15–35-year-old women and is assumed to be 90% effective without leading to a decrease in condom use. Contours represent points of equal PrEP impact. The figure shows that 10%–25% more infections could be averted in the targeted age group when this PrEP strategy covers 30%–60% of women in this group. The population-level effect would be 5%–12% of all infections averted.

The additional benefit of PrEP remains independent of expansion of the national ART program until ART coverage reaches three times its 2010 levels. Beyond this level, which could be reached by 2025 by a UTT strategy with an annual testing and enrollment rate of 20% starting in 2014, the additional benefit of PrEP decreases rapidly to the point of becoming ineffective as the epidemic would be virtually extinct.

The model is based on the premise that heterogeneity, particularly with respect to risk of infection, is structured by age. An interesting policy option could be the targeting of PrEP to narrower age groups. In Fig. 3b PrEP is targeted to 15–25-year-old women and is assumed to be 90% effective without leading to a decrease in condom use. At a given coverage, incremental PrEP impact in the targeted group improves compared to Fig. 3a and the overall impact drops marginally. For example, PrEP averts 12%–27% more infections when 30%–60% of the cohort of women who were 15–25 years old between 2014 and 2025 use PrEP. The population-level effect of PrEP would be less than 9% at 60% targeted coverage.

When PrEP is targeted to 25–35-year-old women, those at highest risk of infection in South Africa (shown in Fig. 3c), the incremental PrEP impact in the targeted group is comparable and the overall impact improves marginally with respect to the scenario depicted in Fig. 3b. Here too it is assumed that PrEP use is 90% effective in preventing infection without leading to a decrease in condom use.

**Figure 5:**
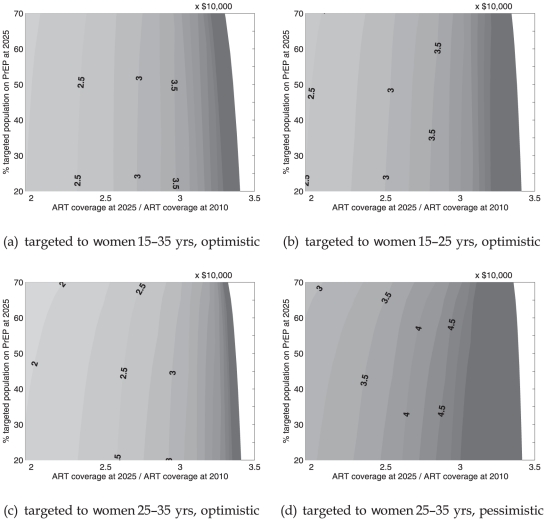
Incremental cost-effectiveness ratio of PrEP: (incremental cost of PrEP)/(additional infections averted due to PrEP). Baseline incidence 0.5% per year at 2025. Contours in intervals of $10,000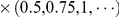. Contours above $50,000 are grayed out.

In Fig. 3d PrEP initiation is targeted to 25–35-year- old women only, is assumed to be 70% effective and decreases condom use among these women by 30% in addition to its decrease with age (Fig. 5 [11]). In this scenario PrEP impact in the 25–35–year–old target group and overall impact is about 25% less than the impact when no condom substitution is assumed.

When baselines incidence is higher there are naturally more infections for PrEP to avert leading to an increase in the effectiveness of PrEP. Comparing Fig. 4a and Fig. 3a shows that at higher baseline incidence 30%–60% PrEP coverage would avert 13%–28% more infections in the targeted group and 7%–16% of all infections. In Fig. 3a the estimates for incidence reductions are 13%–28% in the targeted group and 7%–16% overall. Comparing all scenarios of Fig. 4 and Fig. 3 shows that PrEP would have between 20% and 25% greater impact in targeted groups should higher incidence be acting from 2014–2025 than our baseline model predicts. PrEP effectiveness in averting new infections remains dependent on the increase in ART coverage, but greater ART coverage must be reached before PrEP becomes ineffective.

### Targeted PrEP: cost-effectiveness

From a programmatic point of view, key questions are: what is the cost of each infection averted by a PrEP strategy and at what level of ART coverage will cost-effectiveness arguments still favor its use? As in [12], we perform a simple calculation of the cost per person-years of PrEP per infection averted over the period 2014–2025 for each PrEP strategy.

Fig. 5 shows the incremental cost-effectiveness of PrEP strategies corresponding to those in Fig. 3. PrEP would cost more that $20,000 in all of these scenarios. Targeting PrEP to 25–35-year-old women in an optimistic efficacy scenario (Fig. 5c) would be most cost-effective. The condom substitution scenario depicted in Fig. 5d shows a decrease in const-effectiveness of about 35%–40% relative to Fig. 5c, a scenario where no condom substitution is assumed.

**Figure 6:**
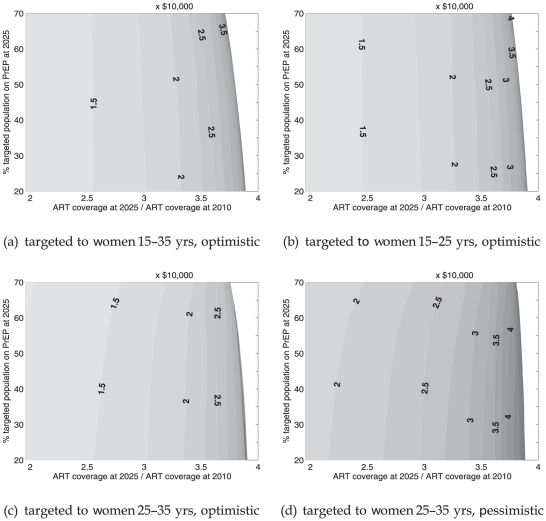
Incremental cost-effectiveness ratio of PrEP: (incremental cost of PrEP)/(additional infections averted due to PrEP). Baseline incidence 0.8% per year at 2025. Contours in intervals of $10,000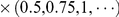. Contours above $50,000 are grayed out.

When baseline incidence is higher and when there are more infections to avert, the cost-effectiveness of PrEP improves significantly. The most cost-effective targeting scenario is shown in Fig. 6c, where PrEP is targeted to 25–35-year-old women when there is a baseline incidence of 0.8% per year at 2025. In this scenario, the cost per infection averted could be as low as $12,000 at low ART coverage.

What these cost scenarios have in common is that an expanding ART program would lead to a dramatic increase in the cost per infection averted with PrEP. It appears that PrEP would have only a window of opportunity to be cost-effective: that is until ART coverage reaches a critical level of roughly three times the coverage in 2010. Naturally there is uncertainty about what the exact critical level of ART will turn out to be, as it is strongly connected to the uncertainty in baseline incidence. However, the existence of this critical level of ART coverage, in terms of rendering PrEP relatively cost-ineffective, is largely independent of baseline incidence, PrEP coverage and PrEP efficacy assumptions.

The WHO-Choice Project and the Commission on Macroeconomics provide guidelines for evaluating the cost-effectiveness of public health interventions in terms of cost per disability-adjusted life years (DALY) saved. In terms of these guidelines, a cost per DALY saved is said to be cost-effective if it is less than three times the gross national income (GNI) per capita, and very cost-effective if it is less than the GNI per capita [39], [40]. The GNI per capita of South Africa is estimated at $5,820 [41]. Therefore, if one HIV infection averted results in 25 DALYs (a conservative estimate which could be obtained using the method outlined in [42]), PrEP would be judged as very cost-effective at a cost of up to 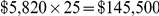 per infection averted, and cost-effective at 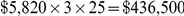 per infection averted. Indeed, most interventions will be found to be highly cost-effective using these guidelines [42]. Clearly, these guidelines cannot locate a meaningful cost-effectiveness threshold on Figs. 5 and 6.

A more useful approach is to compare the cost-effectiveness of PrEP and the number of infections it could potentially avert with that of other interventions (Ch.15 [43]). In the model presented here, a meaningful comparison can only be drawn with respect to ART cost-effectiveness. Our simulations show that, assuming a 20% testing and enrollment rate from 2014 onward, ART alone (i.e. no PrEP) would avert 20% of new infections by 2025 at a cost of $10,000 per infection averted. This 20% annual testing rate is relative to a baseline of current ART expansion, which is driven at present by a testing and enrollment rate closer to 10% per year. Thus, even if annual PrEP does indeed cost four times less than annual ART, expanding ART would still prove more effective in terms of cost per infection averted. While the above-mentioned GNI-based guidelines will not judge PrEP as cost-ineffective, even at high ART coverage levels, it is clear that the cost per infection averted contours become tightly bunched when ART exceeds three times its coverage in 2010. However, we showed in Figs. 2b, 3 and 4 that there could be substantial marginal benefit when PrEP works in tandem with ART to prevent new infections.

## Discussion

How should we compare PrEP and UTT? Are they competing strategies or complementary? What are their expected relative impacts? We should note first that PrEP is a purely preventative strategy, whereas UTT has a dual effect. It is both a treatment and a prevention strategy. Even if PrEP were to prove more cost-effective than UTT as a preventative method, which appears unlikely based on our modelled results, in practice ART coverage would continue to expand as eligibility criteria for treatment are relaxed. These criteria are more based on treatment guidelines than on arguments for the cost-effectiveness of ART as a prevention strategy.

We now turn to matters of impact, coverage and cost of different PrEP strategies, noting the uncertainty in the impact of such factors as HIV incidence when PrEP is introduced, annual cost, targeting strategies and behavior change.

In theory (as suggested by Fig. 2a) non-targeted PrEP coverage would have to be impractically high to have an effect comparable to the effect of UTT on incidence reduction. In order to approach disease eradication, almost all those susceptible to infection must be protected by PrEP. However, Fig. 3a shows that targeting PrEP initiation to 15–35 year-old women would achieve a 10%–25% reduction in new infections by 2025 in the targeted age group. Although optimistic in its assumptions (90% PrEP efficacy, no condom substitution), this scenario indicates that properly managed and targeted PrEP interventions can achieve a non-negligible reduction in incidence.

In each of the targeted PrEP scenarios set out in the section titled “Targeted PrEP: cost-effectiveness”, the cost in person-years of PrEP per infection averted is greater than $20,000 across a range of PrEP coverage (Fig. 5). These estimates appear higher than estimates in [12], where it is reported to be lower than $1,000 in optimistic targeted settings. One explanation for the difference is the assumption of high HIV incidences, e.g. 2.4% per year in 2007 among adults in South Africa, used in [12]. Our estimate, based on data from UNAIDS and ASSA [33] is much lower at 1% per year in 2007 (Fig. 2b, upper solid line) and declines to 0.5% per year by 2025. We created a simulation where baseline incidence declines more gradually to 0.8% per year by 2025, based on condom-use declining by 15% from 2007 onward. The results show that the cost per infection averted could be much lower and closer to $10,000 when ART coverage remains low and incidence turns out to be significantly higher during the period 2014–2025.

The method we used to increase baseline incidence (by relaxing future condom use) causes a rebounding HIV epidemic, and it is not clear that the model can still be used in this way to establish a convincing relationship between baseline incidence, ART coverage and the cost-effectiveness of PrEP. It would be more convincing to fit this model, or similar models, to different generalized HIV epidemics with a range of baseline incidences and HIV epidemics in different stages of retreat.

Vissers et al. [5] found that condom substitution can nullify the benefit of PrEP in certain scenarios (when targeted to a high-risk group at low PrEP coverage). Our analysis shows a smaller impact of condom substitution among targeted PrEP users than was reported in [5], which may also be related to declining incidence in our model. However, note that while condom use in South Africa is relatively high among 15–30-year-old women (Fig. 5 [11]), it decreases exponentially with age (as suggested by DHS surveys in 1998, 2002, 2005, and 2008 and depicted in Fig. 5 [11]). For all women, including those using PrEP, there will be an expected decline in condom use over time based based on our modelling assumptions regarding condom use. At the same time PrEP users would move into age categories of lower sexual activity and lower risk of infection. Risk compensation by 25–35-year-old women using PrEP could lead to a further decrease in condom use over the period 2014–2025, in addition to the above-mentioned decrease-with-age effect, but this on its own will not necessarily result in an overall increase in incidence during the period 2014–2025 (Fig. 3d). Thus in evaluating PrEP programs over long periods of time, one must consider the extra complexity of knowing which part of this decline in condom use can be attributed to risk compensation as opposed to aging.

In all scenarios examined, both the relative cost-effectiveness of PrEP and its impact on incidence would certainly be considerably reduced should UTT be introduced in South Africa shortly after the initiation of a PrEP strategy. Even an increased rate of expansion of the current national ART program would obscure the benefits of PrEP.

The national ART program of South Africa is currently on a scaling-up trajectory where less than 5% of the adult population will receive ART by 2025 while more than 6.5% of adults (which translates to more than 65% of HIV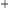 cases) need to receive ART for a significant impact on incidence to result. PrEP could serve as a useful stop-gap control solution until ART coverage is scaled up towards providing UTT-like coverage, after which the epidemic may be substantially controlled in South Africa. This window of opportunity may turn out to be long in resource-limited settings.

It is important to realize that PrEP and ART are only two of several interventions that can be scaled up for cost-effective HIV prevention in South Africa. Programs aimed at commercial sex workers (CSW) and their clients, men who have sex with men (MSM), youth education programs, male-circumcision, to name just a few, all provide safe and very cost-effective prevention (based on cost per DALY guidelines). Although PrEP and expanding ART are significantly more effective, they are also more costly. One of the key messages of the 2010 International AIDS conference was that funding for prevention is levelling off and in some countries decreasing [8]. It may become necessary to determine the most cost-effective package of interventions to deal with a generalized HIV epidemic. (Cost considerations are less of an issue in smaller risk groups, e.g. MSM and CSW.) A follow-up project similar to the Vissers et al. study [5] (which investigated potential PrEP interventions in Botswana, Kenya and India), involving expanding ART programs in different countries with generalized HIV epidemics, would provide invaluable input to formulating comprehensive control strategies.

## Supporting Information

Text S1 Mathematical model: equations and parameter values.
(0.08 MB PDF)
