# Supplementary material for: Evaluating the Cost-Effectiveness of Pre-Exposure Prophylaxis (PrEP) and Its Impact on HIV-1 Transmission in South Africa
Source: PLoS One. 2010 Nov 5;5(11):e13646. doi: 10.1371/journal.pone.0013646 (PMC2974638; doi:10.1371/journal.pone.0013646)
Supplement: Text S1 — Mathematical model: equations and parameter values. (0.08 MB PDF) [file pone.0013646.s001.pdf]

Supplementary material for: Evaluating the cost-effectiveness of pre-exposure prophylaxis (PrEP) and its impact on HIV-1 transmission in South Africa

Carel Pretorius[1], John Stover[1], Lori Bollinger[1], Nicolas Bacaër[2], Brian Williams[3]
1 Futures Institute, Glastonbury, Connecticut, USA
2 IRD (Institut de Recherche pour le Developpement), Bondy, France
3 SACEMA, DST/NRF Centre of Excellence in Epidemiological Modelling and Analysis, Stellenbosch University, Stellenbosch, South Africa
∗ E-mail: cpretorius@futuresinstitute.org

Mathematical model

The mathematical model underlying this analysis is a simple extension of the model developed and parameterized in [1]. Parameter descriptions for this model are given in table S1, while parameter values are listed in Tab. 1 [1]. Parameter description and values related to inclusion of PrEP in the model descriptions are given in table S2.

It consists of only a few compartments which are all structured by age. Throughout the index k refers either to females ($k = f$) or to males ($k = m$). Let:

- $S_k(t, x)$ be the susceptible population of sex k aged x ($x \geq 1$, age x corresponding to those people whose exact age is between $x - 1$ and x) at time t ($t \geq t_0$).
- $P_k(t, x, y)$ be the HIV$_-$ population of sex k aged x at time t that have received PrEP for y years.
- $I'_k(t, x, y)$ be the population of sex k aged x at time t that has been HIV$_+$ for y years ($1 \leq y \leq x$) who are infected and receiving PrEP.
- $I_k(t, x, y)$ be the population of sex k aged x at time t that has been HIV$_+$ for y years ($1 \leq y \leq x$) who are not receiving ART or PrEP.
- $A_k(t, x, y)$ is the HIV$_+$ population of sex k aged x at time t that is receiving ART and that spent y years ($1 \leq y < x$) infected but without treatment.
- $N_k(t, x) = S_k(t, x) + P(t, x) + I'_k(t, x) + I_k(t, x) + A_k(t, x)$ where, with a slight abuse of notation,

$$P_k(t,x) = \sum_{y \leq x} P_k(t,x,y) \ , \quad I'_k(t,x) = \sum_{y \leq x} I'_k(t,x,y)$$
$$I_k(t,x) = \sum_{y \leq x} I_k(t,x,y) \ , \quad A_k(t,x) = \sum_{y < x} A_k(t,x,y)$$

Keeping the notations of [1], set $J_k(t, x) = I_k(t, x) + \varepsilon \left(I'_k(t, x) + A_k(t, x)\right)$. The force of infection is assumed to be

$$\lambda_f(t,x) = 1 - \exp\Big(-p_f\,(1 - c(t,x))\,r(x) \sum_z s(x,z)\,J_m(t,z)/N_m(t,z)\Big)$$
$$\lambda_m(t,x) = 1 - \exp\Big(-p_m \sum_z (1 - c(t,z))\,r(z)\,s(z,x)\,J_f(t,z)/N_m(t,x)\Big)$$

for non PrEP users and

$$\lambda'_f(t,x) = 1-\exp\Big(-p_f\,(1-\varphi)(1-c'(t,x))\,r(x)\sum_z s(x,z)\,J_m(t,z)/N_m(t,z)\Big)$$

$$\lambda'_m(t,x) = 1-\exp\Big(-p_m\sum_z(1-\varphi)(1-c'(t,z))\,r(z)\,s(z,x)\,J_f(t,z)/N_m(t,x)\Big)$$

for PrEP users. Let θ'_k be the PrEP starting rate for people whose age is between $x-1$ and x. The ART starting rate is assumed to be

$$\theta(t,x,y) = h_2(t)\;\rho(x,y)\,\psi/(1-\psi) + h_3(t)\;\tau \tag{1}$$

Notice that under the current ART program ($h_2 = 1$ and $h_3 = 0$), HIV_+ people are subject to two competing risks: the risk of dying at a rate $\rho(x,y)$, and the risk (the chance) of starting ART at a rate $\rho(x,y)\,\psi/(1-\psi)$. Thus a fraction ψ starts ART.

The rate at which PrEP individuals start ART is assumed to be higher:

$$\theta^*(t,x,y) \quad = \quad \nu\,\theta(t,x,y)\;\nu > 1$$

to account for the likelihood that PrEP users will undergo regular screening and thus be more likely to enroll for ART.

Our assumptions regarding the initial condition $t = t_0$ are as follows. For all $1 \le x \le \omega$, we assume that $S_k(t_0,x) = N_k(t_0,x)$ except that $S_m(t_0,x_0) = N_m(t_0,x_0) - 1$. For all $1 \le y \le x \le \omega$, we assume that $I_k(t_0,x,y) = 0$ except that $I_m(t_0,x_0,1) = 1$. Finally, $A_k(t_0,x,y) = 0$ for all $1 \le y < x \le \omega$.

For $t \ge t_0$ and $1 \le x \le \omega-1$, the susceptible population is given by

$$\begin{aligned}
S_k(t+1,1) &= b(t)\,(1-\pi(t)),\\
S_k(t+1,x+1) &= (1-\mu_k(x))(1-\lambda_k(t,x))(1-\theta'_k(t,x))S_k(t,x)\\
&\quad + (1-\mu_k(x))\,(1-\lambda'_k(t,x))\,\phi'\,P_k(t,x)
\end{aligned}$$

The PrEP receiving population is given for $t \ge t_0$ and $1 \le y \le x$ by

$$\begin{aligned}
P_k(t+1,x+1,1) &= (1-\mu_k(x))\,(1-\lambda_k(t,x))\,\theta'_k(t,x)\,S_k(t,x),\\
P_k(t+1,x+1,y+1) &= (1-\mu_k(x))\,(1-\lambda'_k(t,x))\,(1-\phi')\,P_k(t,x,y)
\end{aligned}$$

The PrEP receiving population who are infected is given for $t \ge t_0$ and $1 \le y \le x \le \omega-1$ by

$$\begin{aligned}
I'_k(t+1,x+1,1) &= (1-\mu_k(x))\,\lambda'_k(t,x)\,(1-\phi')\,P_k(t,x)\\
&\quad + (1-\mu_k(x))\lambda_k(t,x)\theta'_k(t,x)\,S_k(t,x)\\
I'_k(t+1,x+1,y+1) &= (1-\mu_k(x))\,(1-\rho(x,y))\,(1-\phi'_+)\,(1-\theta^*(t,x,y))\,I'_k(t,x,y)
\end{aligned}$$

Here $\phi'_+ \ge \phi$ is the rate at which HIV_+ PrEP users will discontinue PrEP use. The infected population without treatment is given for $t \ge t_0$ and $1 \le y \le x \le \omega-1$ by

$$\begin{aligned}
I_k(t+1,1,1) &= b(t)\,\pi(t),\\
I_k(t+1,x+1,1) &= (1-\mu_k(x))\,\lambda_k(t,x)\,(1-\theta'_k(x))\,S_k(t,x),\\
&\quad + (1-\mu_k(x))\,\lambda'_k(t,x)\,\phi'\,P_k(t,x,y)\\
I_k(t+1,x+1,y+1) &= (1-\mu_k(x))(1-\rho(x,y))(1-\theta(t,x,y))\,I_k(t,x,y)\\
&\quad + (1-\mu_k(x))\,\phi\,(1-\sigma(y))\,A_k(t,x,y)\\
&\quad + (1-\mu_k(x))(1-\rho(x,y))\,\phi'_+\,(1-\theta^*(t,x,y))\,I'_k(t,x,y)
\end{aligned}$$

The ART-treated population is given for $t \geq t_0$ and $1 \leq y \leq x \leq \omega - 1$ by

$$\begin{aligned} A_k(t+1, x+1, y) &= (1-\mu_k(x))\,(1-\phi)\,(1-\sigma(y))\,A_k(t,x,y) \\ &\quad + (1-\mu_k(x))(1-\rho(x,y))\,\theta(t,x,y)\,I_k(t,x,y) \\ &\quad + (1-\mu_k(x))(1-\rho(x,y))(1-\phi'_+)\theta^*(t,x,y)\,I'_k(t,x,y) \end{aligned}$$

setting $A_k(t,1,1) = 0$ for all t for convenience.

Acknowledgments

References

1. Bacaer N, Pretorius C, Auvert B (2010) An age-structured model for the potential impact of generalized access to antiretrovirals on the South African HIV epidemic. Bull Math Biol doi:10.1007/s11538-010-9535-2.

Tables

Table 1. Notations and parameter description. "M2C" stands for mother-to-child, "prob." for probability.

k	sex (female or male)
t_0	year of introduction of HIV
t	time, $t \geq t_0$
ω	maximum age considered
x	age, $1 \leq x \leq \omega$
y	time since infection without ART
x_0	age of first infected woman
$b(t_0)$	annual male (and female) births at $t = t_0$
p_f	HIV transmission prob. (man to woman)
p_m	HIV transmission prob. (woman to man)
q_0	M2C transmission prob.
q_1	M2C transmission prob. with PMTCT
ε	relative infectiousness of people on ART
ϕ	ART drop-out
τ	annual proportion tested for HIV
$N_k(t_0, x)$	age pyramid at $t = t_0$
$\mu_k(x)$	death rate if HIV_-
$b(t)/b(t_0)$	changing birth rate
$\beta(x)$	normalized female fertility
u	under-reporting of male sexual partners
$u\, r(x)$	reported turnover of male sexual partners
$s(x, y)$	choice of male sexual partner
$c(t, x)$	condom use
$\rho(x, y)$	AIDS mortality
$\rho_1(y)$	adult AIDS mortality
$\sigma(y)$	mortality under ART
$h_1(t)$	access to PMTCT
$h_2(t)$	access to current ART program
$h_3(t)$	access to the "test and treat" strategy
ψ	proportion starting ART in current program

Table 2. Extension to notations and parameters used in [1]. PrEP parameters used in Section: "Universal PrEP and UTT: comparative impact"

Notational parameters	
k: sex (female or male)	f or m
t_0: year of introduction of HIV	
t: time	$t \geq t_0$
ω: maximum age considered	
x: age	$1 \leq x \leq \omega$
y: disease duration	$1 \leq y \leq \omega$
PrEP sub-model	
θ'_k: access to PrEP	20% per year
$\theta(t,x,y)$: access to ART for non-PrEP users	eq. 1
$\theta^* = \nu\,\theta$: access to ART for PrEP users	$\nu = 1.5$
φ: efficacy of PrEP	90%
ϕ': PrEP drop-out	1.5% per year
ϕ'_+: PrEP discontinuation rate	100% per year
$c'_k(t,x)$: condom substitution for those using PrEP	0%
